# Identification of Atrial Transmural Conduction Inhomogeneity Using Unipolar Electrogram Morphology

**DOI:** 10.3390/jcm13041015

**Published:** 2024-02-09

**Authors:** Lu Zhang, Mathijs S. van Schie, Hongxian Xiang, Rongheng Liao, Jiahao Zheng, Paul Knops, Yannick J. H. J. Taverne, Natasja M. S. de Groot

**Affiliations:** 1 Department of Cardiology, Erasmus Medical Center, 3015GD Rotterdam, The Netherlandsj.zheng.1@erasmusmc.nl (J.Z.); n.m.s.degroot@erasmusmc.nl (N.M.S.d.G.); 2 Translational Cardiothoracic Surgery Research Lab, Department of Cardiothoracic Surgery, Erasmus Medical Center, 3015GD Rotterdam, The Netherlands; 3 Signal Processing Systems, Department of Microelectronics, Faculty of Electrical Engineering, Mathematics and Computer Sciences, Delft University of Technology, 2628CD Delft, The Netherlands

**Keywords:** simultaneous endo-epicardial mapping, transmural conduction block, endo-epicardial delay, electrograms, sinus rhythm

## Abstract

(1) Background: Structural remodeling plays an important role in the pathophysiology of atrial fibrillation (AF). It is likely that structural remodeling occurs transmurally, giving rise to electrical endo-epicardial asynchrony (EEA). Recent studies have suggested that areas of EEA may be suitable targets for ablation therapy of AF. We hypothesized that the degree of EEA is more pronounced in areas of transmural conduction block (T-CB) than single-sided CB (SS-CB). This study examined the degree to which SS-CB and T-CB enhance EEA and which specific unipolar potential morphology parameters are predictive for SS-CB or T-CB. (2) Methods: Simultaneous endo-epicardial mapping in the human right atrium was performed in 86 patients. Potential morphology parameters included unipolar potential voltages, low-voltage areas, potential complexity (long double and fractionated potentials: LDPs and FPs), and the duration of fractionation. (3) Results: EEA was mostly affected by the presence of T-CB areas. Lower potential voltages and more LDPs and FPs were observed in T-CB areas compared to SS-CB areas. (4) Conclusion: Areas of T-CB could be most accurately predicted by combining epicardial unipolar potential morphology parameters, including voltages, fractionation, and fractionation duration (AUC = 0.91). If transmural areas of CB indeed play a pivotal role in the pathophysiology of AF, they could theoretically be used as target sites for ablation.

## 1. Introduction

Structural remodeling plays an important role in the pathophysiology of atrial fibrillation (AF) [1,2]. Although there is still ongoing debate on the mechanism underlying AF, several main theories have been described over the past few years, as recently discussed in the 2023 ACC/AHA/ACCP/HRS Guideline for the Diagnosis and Management of Atrial Fibrillation [3]. Key elements in the pathophysiology of persistent AF are areas of conduction block (CB) and electrical asynchrony between the endo- and epicardial layers [2,4,5,6,7,8]. Prior mapping studies demonstrated that even during sinus rhythm, CB is more prevalent in patients with AF compared to patients without AF [9]. The increase in conduction disorders can be explained by a higher degree of structural remodeling consisting of, e.g., interstitial fibrosis and proteostasis derailment, which occurs not only at the endo- or epicardium but also transmurally [10,11,12].

Previous simultaneous endo-epicardial mapping studies demonstrated that lines of CB can be located at either the endo- or epicardium only (single-sided (SS-CB)) or at both layers (transmural CB (T-CB)) [13]. Areas of CB give rise to abnormalities in potential morphology, including a reduction in voltage and fractionation [14,15,16]. It is likely that CB enhances endo-epicardial delay (EED), although this relationship has so far not been confirmed. Recently, it has been suggested that AF can be treated by ablation therapy of areas of endo-epicardial asynchrony [17]. We hypothesize that the degree of EED is more pronounced in areas of T-CB than SS-CB at either the endo- or epicardium. Hence, we investigated the degree to which SS-CB and T-CB enhance EED and whether unipolar potential morphology associated with the different types of CB is predictive for T-CB.

## 2. Materials and Methods

### 2.1. Study Population

Eighty-six patients (68 (79%) male, age: 67 (61–72) years) undergoing cardiac surgery for coronary artery disease (*n* = 43), heart valve disease (*n* = 42), or arrhythmia surgery (*n* = 1) in the Erasmus Medical Center Rotterdam were included. The study was conducted according to the guidelines of the Declaration of Helsinki and approved by the Institutional Medical Ethics Committee of Erasmus MC (MEC2015-373, 29 October 2015). Written informed consent was obtained from all patients. Patient characteristics (e.g., age, medical history, and cardiovascular risk factors) were obtained from each patient’s medical record. Patients with hemodynamic instability, atrial paced rhythm, previous open cardiac surgery, severe liver or renal failure, or severely impaired left ventricular function were excluded from the study. 

### 2.2. Simultaneous Endo-Epicardial Mapping of the RA 

The methodology of simultaneous endo-epicardial high-resolution mapping has been described in detail previously [18]. The mapping procedure was performed with two electrode arrays, each of which had 128 (8 × 16) unipolar electrodes with a 0.45 mm diameter and 2 mm interelectrode spacing. The arrays were secured on two flexible spatulas and positioned directly across from the right atrial (RA) wall. A temporal bipolar epicardial pacemaker wire was connected to the free wall as a reference electrode following heparinization and arterial cannulation. The indifferent electrode was anchored to the subcutaneous tissue of the thoracic cavity. After an incision into the RA appendage was made, the spatula designated as the endocardial electrode array was inserted, and the RA was subsequently closed with a purse-string suture. 

Three separate areas on the superior, mid, and inferior free walls of the RA underwent simultaneous endo-epicardial mapping, as shown in Supplementary Figure S1. After the insertion of the final row of electrodes in the array, the endocardial spatula was inserted at least 1.5 cm deeper into the RA to prevent overlap of the mapping area close to the incision site. During stable sinus rhythm, electrograms (EGMs) were collected for 5 s. Surface ECG leads, a calibration signal of 2 mV and 1000 ms, and a bipolar reference EGM were all recorded. After sampling (1 kHz), amplification (gain 1000), filtering (bandwidth 0.5–400 Hz), and analog-to-digital conversion, data were saved on a hard drive (16 bits).

### 2.3. Mapping Data Analysis 

Mapping data were evaluated using dedicated custom-made Python 3.8 software. Color-coded activation maps were created by annotating the steepest negative slope of a unipolar potential as local activation time (LAT). All annotations were manually reviewed by two investigators. As illustrated in Supplementary Figure S1, unipolar potentials were divided into four distinct groups according to their morphology, including (1) single potentials (SPs) consisting of a single negative deflection; (2) double potentials (DPs) subdivided into short double potentials (SDPs) and long double potentials (LDPs) containing two deflections separated by <15 ms and ≥15 ms, respectively; and (3) fractionated potentials (FPs) consisting of ≥3 deflections. Fractionation duration (FD) was defined as the time difference between the first and last deflection of non-single potentials [19]. 

The peak-to-peak amplitude of the steepest deflection was defined as potential voltage, and low-voltage areas (LVAs) were defined as sites from which potentials with voltages < 1.0 mV were recorded. Endo-epicardial dissociation (EED) was defined as LAT differences between the endo- and epicardial layers as previously described, and EEA as an EED > 15 ms [13]. The total amount of EEA for each patient was calculated as the proportion (EEA%) of the total mapping area. 

Local CB was defined as an LAT difference of ≥12 ms between adjacent electrodes (Supplementary Figure S1), which corresponds to an effective conduction velocity of <19 cm/s as previously described [8]. SS-CB was defined as CB confined to only the endo- or epicardium and was referred to as SS-CB_endo_ or SS-CB_epi_, respectively. T-CB was defined as the presence of CB lines in opposite endocardial and epicardial mapping sites. In addition, all electrophysiological parameters were separately measured at electrodes adjacent to each line of CB.

### 2.4. Statistical Analysis

Continuous variables with normally distributed distributions were displayed as mean and standard deviation (SD), whereas skewed data were given as median (25th, 75th percentile). The Mann–Whitney U test or the Kruskal–Wallis test was employed to compare differences across groups. The chi-squared test was used to compare categorical data provided as numbers and percentages. IBM SPSS Statistics version 26 (IBM Corp, Armonk, NY, USA) and Python 3.8 were used for statistical analysis.

## 3. Results

### 3.1. Study Population 

The characteristics of the population (*n* = 86, age 67 (61–72) years, 68 male (79.1%)) are listed in Table 1. A history of AF was present in 37 (43.0%) patients.

### 3.2. Mapping Data

A total of 1641 (19 ± 7.5 per patient) sinus rhythm beats were recorded (cycle length: 876 ± 190 ms). A total of 162,443 potentials were recorded from both the endo- and epicardium (total: 324,886). Unipolar potential voltages at the endo- and epicardium were 4.65 (3.15–6.52) mV and 7.01 (5.34–8.35) mV, respectively (*p* < 0.001, r = 0.681). EEA was present in 5.8% of all mapping areas.

### 3.3. Prevalence of Transmural Conduction Block

**Figure 1:**
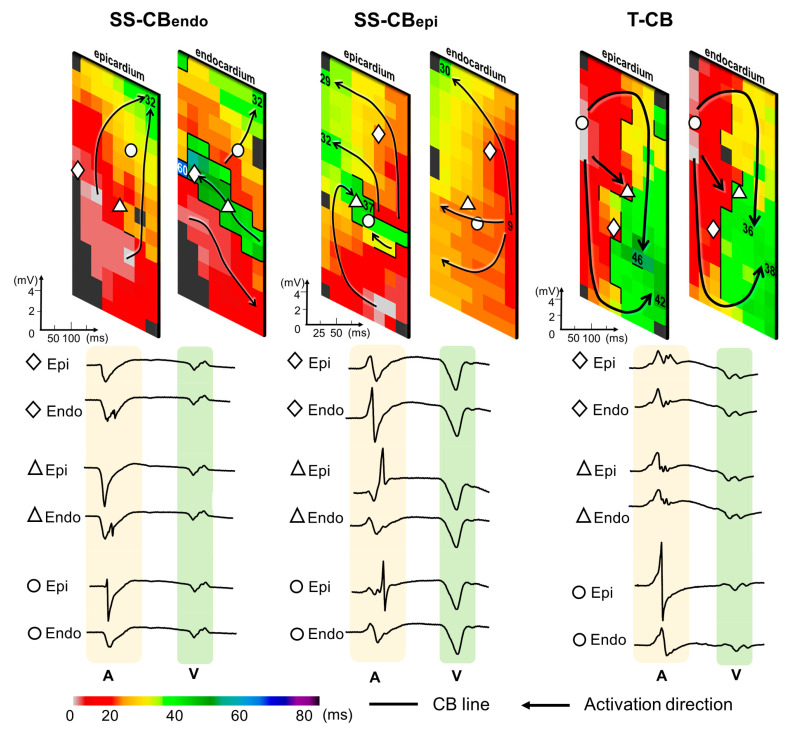
Examples of color-coded activation maps demonstrating SS-CB_endo_, SS-CB_epi_, and T-CB areas constructed by simultaneous endo-epicardial mapping. Thick black lines indicate lines of CB and black arrows show the main activation wavefront trajectories. The numbers on the electrodes indicate the local activation time. Opposite endo- and epicardial potentials are depicted below the activation maps. SS-CB_endo_ = endocardial single-sided conduction block; SS-CB_epi_ = epicardial single-sided conduction block; T-CB = transmural conduction block; epi = epicardial; endo = endocardial; CB = conduction block; A = atrial potential; V = ventricular far-field.

Figure 1 illustrates three opposite endo- and epicardial activation maps. These maps show SS-CB_endo_, SS-CB_epi_, and T-CB, as indicated by the thick black lines. T-CB was present at 4301 recording sites, which was 26.4% of the total amount of CB at the endocardium (*n* = 16,307) and 37.1% of that at the epicardium (*n* = 11,594). 

**Figure 2:**
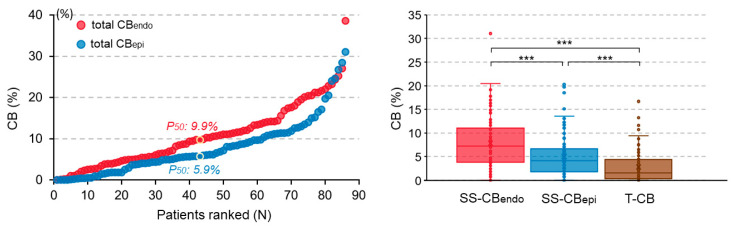
The (**left panel**) shows the amount of total CB at the endo- and epicardium for each individual patient; patients are ranked according to an increasing number of total CB (%). The (**right panel**) compares the amount of SS-CB_endo_, SS-CB_epi_, and T-CB for each patient. *** means *p* < 0.001. SS-CB_endo_ = endocardial single-sided conduction block; SS-CB_epi_ = epicardial single-sided conduction block; T-CB = transmural conduction block; CB = conduction block.

The left panel of Figure 2 demonstrates the amount of total CB at the endo- and epicardium for each individual patient. Areas of CB were found in 85 (98.8%) and 82 (95.3%) patients at the endo- and epicardium, respectively; the corresponding proportions of CB areas ranged from 0% to 38.6% and 0 to 31.0%. CB areas were found more frequently at the endocardium than at the epicardium (9.9 (4.9–14.2) % vs. 5.9 (3.1–11.1) %, *p* < 0.001). 

The right panel of Figure 2 illustrates the amount of SS-CB_endo_, SS-CB_epi_, and T-CB for each patient separately. SS-CB was more frequently found at the endocardium than epicardium (7.25 (3.8–10.97) % vs. 4.11 (1.81–6.49) %, *p* < 0.001), and areas of SS-CB at either the endo- or epicardium occurred more frequently than T-CB (1.51 (0.35–4.2) %, *p* < 0.001).

### 3.4. Relation between Different Types of CB and EED

As listed in Table 2, EED was more pronounced in CB areas than non-CB areas (10 (5–17) ms vs. 3 (2–5) ms, *p* < 0.001), and the largest degree of EED was found in T-CB areas (T-CB: 13 (6–21) ms vs. SS-CB_endo_: 9 (4–15) ms and SS-CB_epi_: 8 (4–15) ms, *p* < 0.001). 

### 3.5. T-CB and Unipolar Potential Voltages

**Figure 3:**
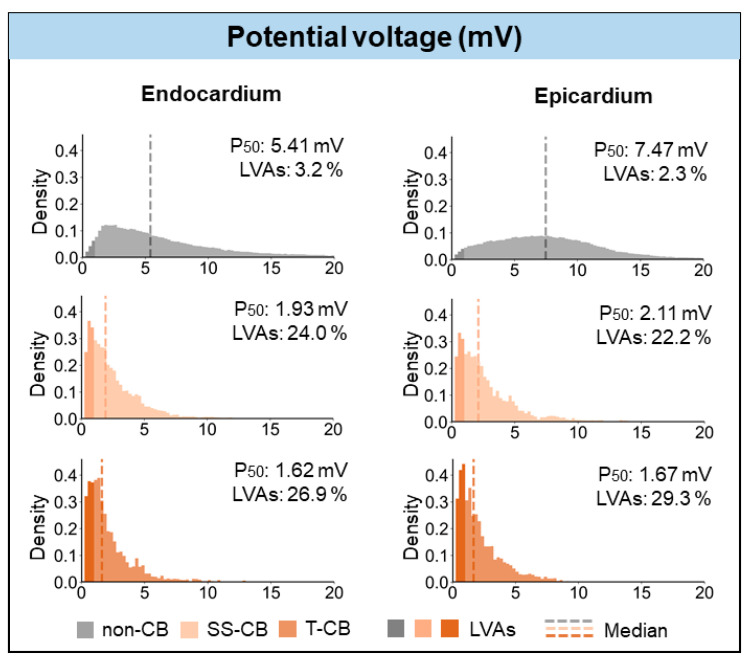
Histograms of the relative frequency distribution of potential voltages in non-CB, SS-CB_endo/epi_, and T-CB areas at endocardium (**left panel**) and epicardium (**right panel**). Grey, peach, and brown colors represent potential voltages in the non-CB, SS-CB, and T-CB areas, respectively. Darker colors highlight LVAs. Dashed lines indicate median values. LVA = low-voltage area; SS-CB = single-sided conduction block; T-CB = transmural conduction block.

Figure 3 shows unipolar potential voltages in areas without CB and areas with SS-CB_endo_, CB_epi_, or T-CB. In areas of T-CB measured from either the endo- or epicardium, unipolar potential voltages were lower compared to those in areas of SS-CB at both the endocardium (1.62 (0.95–2.86) mV vs. 1.93 (1.03–3.47) mV, *p* < 0.001) and epicardium (1.67 (0.9–3.0) mV vs. 2.11 (1.1–3.75) mV, *p* < 0.001).

LVAs were rare at both the endo- and epicardial layers but were most prevalent in T-CB areas (endocardium: T-CB: 26.9% vs. SS-CB_endo_: 24.0% vs. non-CB areas: 3.2%, *p* < 0.001; epicardium: T-CB: 29.3% vs. SS-CB_epi_: 22.2% vs. non-CB areas: 2.3%, *p* < 0.001).

### 3.6. T-CB and Unipolar Potential Morphology

**Figure 4:**
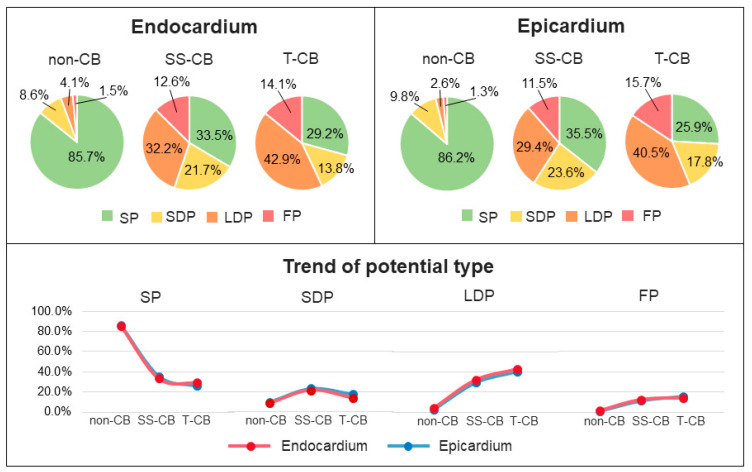
Relationship between CB and potential types. The upper panel shows the composition of various potential types in non-CB, SS-CB, and T-CB areas at the endocardium (**left**) and epicardium (**right**). The lower panel illustrates trends from non-CB to SS-CB_endo/epi_ to T-CB areas for each potential type at the endocardium (red dots) and epicardium (blue dots). SS-CB = single-sided conduction block; T-CB = transmural conduction block; SP = single potentials; SDP = short double potentials; LDP = long double potentials; FP = fractionated potentials.

Figure 4 shows the proportion of different potential types (SP, SDP, LDP, and FP) in mapping areas with SS-CB or T-CB at the endo- and epicardium separately. At the endocardium, T-CB areas contained more LDP and FP and less SP and SDP compared to SS-CB_endo_ areas (SP: 29.2% vs. 33.5%, SDP: 13.8% vs. 21.7%, LDP: 42.9% vs. 32.2%, FP: 14.1% vs. 12.6%). Likewise, at the epicardium, T-CB areas also contained more LDP and FP and less SP and SDP compared to SS-CB_epi_ areas (SP: 25.9% vs. 35.6%, SDP: 17.8% vs. 23.6%, LDP: 40.5% vs. 29.4%, FP: 15.7% vs. 11.5%). 

**Figure 5:**
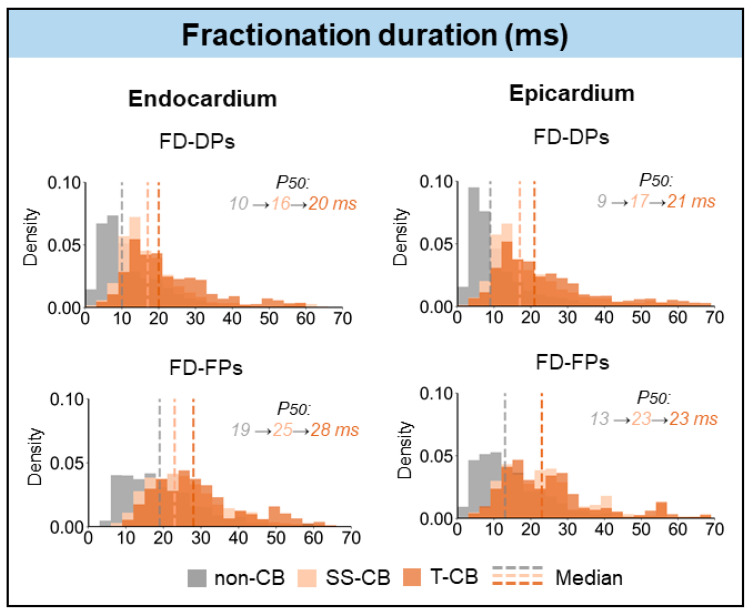
Relative frequency distribution of FD in non-CB, SS-CB_endo/epi_, and T-CB areas of the endocardium (**left**) and epicardium (**right**). Grey, peach, and brown colors represent FD in the non-CB, SS-CB, and T-CB areas, respectively. Dashed lines indicate median values. SS-CB = single-sided conduction block; T-CB = transmural conduction block; FD = fractionation duration; DP = double potentials; FP = fractionated potentials.

Figure 5 demonstrates the FD of DP and FP for the different categories of CB at the endo- and epicardium separately. FD of DP was most prolonged in areas of T-CB at both the endo- and epicardium (LDP: endocardium: T-CB: 20 (15–30) ms vs. SS-CB_endo_: 16 (12–22) ms, *p* < 0.001; epicardium: T-CB: 21 (14–31) ms vs. SS-CB_epi_: 17 (12–25) ms, *p* < 0.001). FD of FP was only prolonged in T-CB areas at the endocardium (T-CB: 28 (20–36) ms vs. SS-CB_endo_: 25 (19–33) ms, *p* < 0.001).

### 3.7. Prediction of T-CB Areas

**Figure 6:**
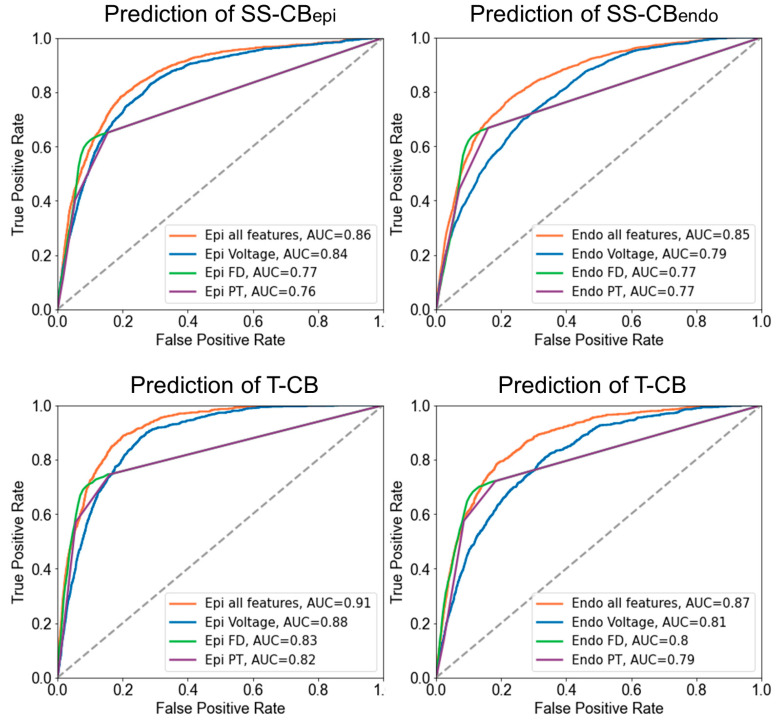
ROC curves of the prediction of SS-CB_endo/epi_ (**upper panel**) and T-CB areas (**lower panel**) based on EGM features recorded from the endo- or epicardium. SS-CB_endo_ = endocardial single-sided conduction block; SS-CB_epi_ = epicardial single-sided conduction block; T-CB = transmural conduction block; FD = fractionation duration; PT = potential type.

Figure 6 demonstrates the receiver operating characteristic (ROC) curves of the accuracy of identifying SS-CB or T-CB areas using the potential morphology parameters. When using a single potential variable, unipolar potential voltages were the optimal parameter for predicting both SS-CB and T-CB. However, the prediction of the different types of CB areas was most accurate when combining all three parameters (endocardium: SS-CB: AUC_endo_ = 0.85, T-CB: AUC_endo_ = 0.87, epicardium: SS-CB: AUC_epi_ = 0.86, T-CB: AUC_epi_ = 0.91). All epicardial potential morphology parameters were most accurate for the prediction of T-CB, and the epicardial potential voltage had the highest predictive value for T-CB areas (AUC = 0.88).

## 4. Discussion

Electrical conduction delay between the endo- and epicardium is mostly affected by the presence of transmural areas of conduction block. These areas of T-CB can be most accurately predicted by combining epicardial potential morphology parameters, including voltages, fractionation, and FD.

### 4.1. Endo-Epicardial Asynchrony Related to Transmural Conduction Block

Prior mapping studies demonstrated that focal waves and long lines of CB are key elements in AF persistence [8,20]. However, focal waves can only arise in the presence of EEA. Indeed, electrical asynchrony between the epi- and endocardial layers occurs more frequently in patients with persistent types of AF [4]. A previous simultaneous endo-epicardial mapping study showed that even during SR, a certain degree of EEA is present and may be more pronounced in patients with AF compared to patients without atrial tachyarrhythmias [13]. However, the relation between (different types of) CB and asynchrony between the endo- and epicardial layer has so far never been examined. In areas of SS-CB, a certain degree of endo-epicardial conduction delay was observed which further increased in T-CB areas. This may be explained by more severe structural remodeling such as deposition of fibrotic tissue or loss of cell-to-cell communications, which enhance discontinuities between the endo- and epicardium. This in turn results in an increase in the length of the activation pathway and hence prolonged differences in activation time between both layers [4].

### 4.2. Potential Morphology as Indicator of Transmural Conduction Block

The structure of the atrial wall has a considerable impact on elements of unipolar potential morphology such as potential voltages and fractionation [16,21,22]. It is assumed that LVAs may be surrogate indicators of arrhythmogenic substrates [23,24,25]. Indeed, in the present study, we showed that unipolar potential voltages were particularly lower in areas of T-CB compared to SS-CB, which confirmed the observations of Van Schie et al., who demonstrated that unipolar potential voltages were lower near lines of CB [26]. By correlating mapping data with the histology of atrial biopsies, the relation between low voltage potentials and structural changes such as fibrotic depositions, increases in intercellular space, and myofibrillar loss has been confirmed [27]. In particular, voltages of epicardial potentials are related to T-CB, which may be explained by the considerably larger epicardial than endocardial potentials. Consequently, a moderate degree of remodeling may particularly affect the endocardial potential voltages as they are smaller than epicardial potential voltages, whereas a severe degree of remodeling will also affect the larger epicardial potential voltages.

As low-voltage areas commonly contain prolonged FPs and LDPs around lines of CB, they are also abundant in areas of T-CB. Hence, the combination of all three potential morphology parameters (potential voltages, fractionation, and FD) is most accurate in identifying areas of T-CB.

### 4.3. Clinical Implications

In the present study, we demonstrated that epicardial potential morphology parameters are particularly valuable in predicting areas of T-CB. In a previous mapping study, Van Schie et al. also demonstrated that, in particular for voltage mapping, an epicardial approach is favored for identifying low-voltage areas [28]. However, low-voltage areas frequently occurred only at either the endo- or epicardium alone and were not visible when recording from one side only. Although endocardial mapping is more standardly used in clinical practice, there is an increasing use of a so-called hybrid procedure for more persistent types of AF [29,30,31]. Using this hybrid approach, epicardial access would be possible and can more easily be performed. Using the suggested potential morphology parameters, areas of T-CB can thus be targeted with these procedures, which may eventually improve ablation outcomes. Recently, Tung et al. described four cases in which endo-epicardial mapping of the left atrium during AF showed a significant degree of EEA and areas of T-CB [17]. It was suggested that ablation therapies that achieve complete transmurality may be necessary to specifically direct treatment at the epicardium. However, future studies should first reveal whether areas of T-CB play a pivotal role in the pathophysiology of AF.

### 4.4. Limitations

As we performed mapping in humans during cardiac surgery, we could not correlate mapping data with histological analysis of the right atrial free wall. We assumed that opposite endo-epicardial CB lines are representative of T-CB lines, but slow intramural zones of conduction could still be present. We only regarded exact opposite lines of CB as T-CB, but we cannot exclude that more oblique-orientated CB lines are also T-CB lines. Due to concerns regarding the possible presence of air embolisms in the left atrium, only the RA free wall was routinely investigated. 

## 5. Conclusions

Electrical conduction delay between the endo- and epicardium is mostly affected by the presence of transmural areas of conduction block. Areas of transmural conduction block can be most accurately predicted by combining epicardial potential voltages, fractionation, and fractionation duration. Hence, we have identified potential features that can be used to locate T-CB areas which in turn are indicative of areas of endo-epicardial conduction delay. If these areas play a pivotal role in the pathophysiology of AF, they could theoretically be used as target sites for ablation.

## Figures and Tables

**Table 1:** Baseline characteristics.

Baseline Characteristics	
Patients	86
Male	68 (79.1%)
Age (years)	67 (61–72)
BMI (kg/m^2^)	27.9 (24.8–31.0)
Underlying heart disease	
-iHD	43 (50.0%)
-vHD	22 (25.6%)
-cHD	20 (23.2%)
History of AF	37 (43.0%)
-Paroxysmal	31 (36.0%)
-Persistent	4 (4.7%)
-Longstanding persistent	2 (2.3%)
Cardiovascular risk factors	
Hypertension	54 (62.8%)
Hypercholesterolemia	46 (53.5%)
Diabetes mellitus	28 (32.6%)
Left ventricular function	
-Mild impairment (>50% LVEF ≥ 40%)	12 (14.0%)
-Moderate impairment (LVEF 30–39%)	10 (11.6%)
-Severe impairment (LVEF < 30%)	1 (1.2%)
Antiarrhythmic drugs	
-Class I	1 (1.2%)
-Class II	59 (68.6%)
-Class III	6 (7.0%)
-Class IV	6 (7.0%)

Values are presented as *n* (%) or median (interquartile ranges). BMI = body mass index; iHD = ischemic heart disease; vHD = valvular heart disease; cHD = combined heart disease; AF = atrial fibrillation; LVEF = left ventricular ejection fraction.

**Table 2:** EGM characteristics in endo- and epicardial SS-CB and T-CB areas.

	SS-CB_endo/epi_	T-CB	*p*-Value
	Median (IQR)	Median (IQR)	
Endocardium			
Voltage (mV)	1.93 (1.03–3.47)	1.62 (0.95–2.86)	<0.001
EED (ms)	9 (4–15)	13 (6–21)	<0.001
FD (ms)			
FD-DP	16 (12–22)	20 (15–30)	<0.001
FD-FP	25 (19–33)	28 (20–36)	<0.001
Epicardium			
Voltage (mV)	2.11 (1.1–3.75)	1.67 (0.9–3.0)	<0.001
EED (ms)	8 (4–15)	13 (6–21)	<0.001
FD (ms)			
FD-DP	17 (12–25)	21 (14–31)	<0.001
FD-FP	23 (15–29)	23 (16–31)	0.392

EED = endo-epicardial delay; FD = fractionation duration; DP = double potentials; FP = fractionated potentials; SS-CB_endo/epi_ = endo- or epicardial single-sided conduction block; T-CB = transmural conduction block.

## Data Availability

The data underlying this article will be shared on reasonable request to the corresponding author.

## Supplementary Materials

The following supporting information can be downloaded at https://www.mdpi.com/article/10.3390/jcm13041015/s1: Figure S1: A. Simultaneous endo-epicardial mapping of the right atrium. B. Activation map with CB lines. C. The unipolar recording of single electrode types.
