# Supplementary material for: Identification of Atrial Transmural Conduction Inhomogeneity Using Unipolar Electrogram Morphology
Source: J Clin Med. 2024 Feb 9;13(4):1015. doi: 10.3390/jcm13041015 (PMC10889286; doi:10.3390/jcm13041015)
Supplement: Supplementary file 1 [file jcm-13-01015-s001.zip › jcm-2824398-supplementary.pdf]

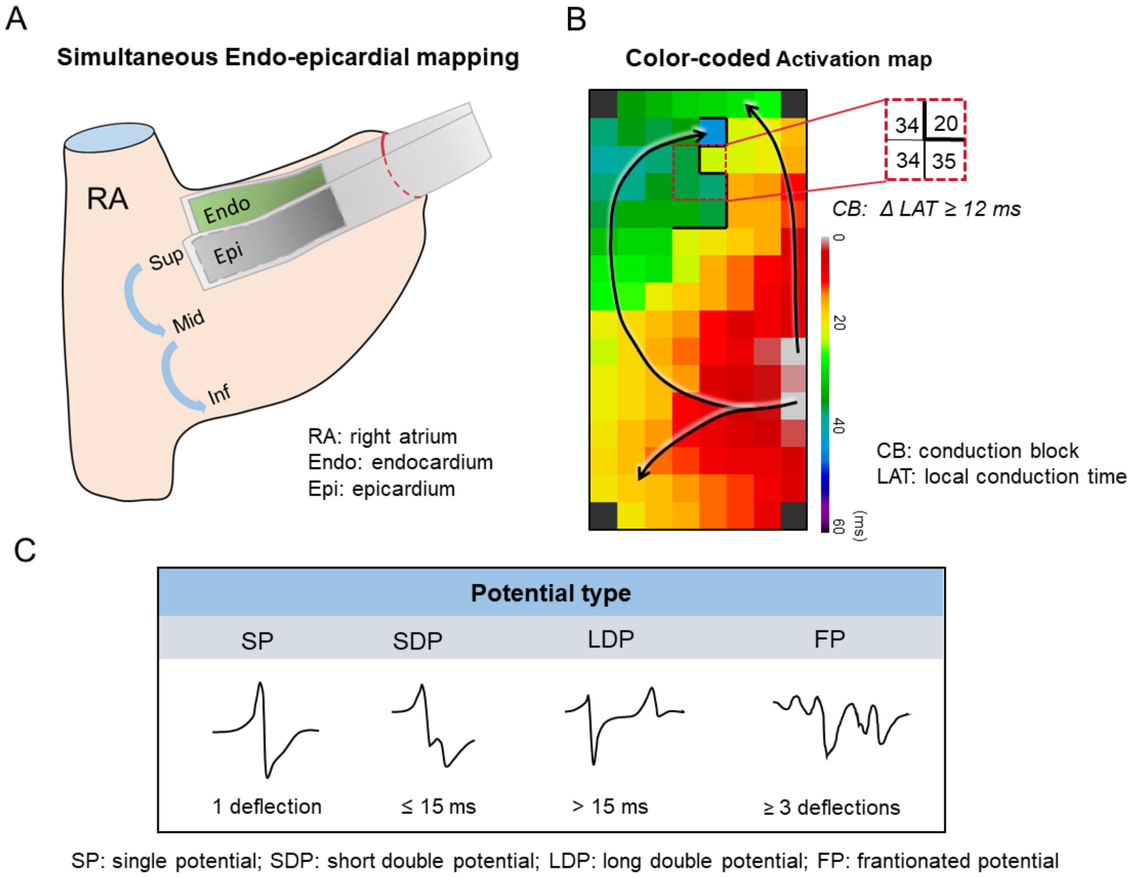

Figure S1. Panel A: simultaneous endo-epicardial mapping procedure covering three sites at the RA (superior, middle and inferior). Panel B: color-coded activation map demonstrating a line of CB which are indicated by thick black lines. Panel C: various unipolar potential types. RA = right atrium; endo = endocardium; epi = epicardium; CB = conduction block; LAT = local activation time; SP = single potential; SDP = short double potential; LDP = long double potential; FP = fractionated potential.
